# The filter of choice: filtration method preference among injecting drug users

**DOI:** 10.1186/1477-7517-8-20

**Published:** 2011-08-22

**Authors:** Lenneke Keijzer, Elliot Imbert

**Affiliations:** 1 Apothicom, 52 Avenue Edison, Paris, 75013, France

## Abstract

**Background:**

Injection drug use syringe filters (IDUSF) are designed to prevent several complications related to the injection of drugs. Due to their small pore size, their use can reduce the solution's insoluble particle content and thus diminish the prevalence of phlebitis, talcosis.... Their low drug retention discourages from filter reuse and sharing and can thus prevent viral and microbial infections. In France, drug users have access to sterile cotton filters for 15 years and to an IDUSF (the Sterifilt^®^) for 5 years. This study was set up to explore the factors influencing filter preference amongst injecting drug users.

**Methods:**

Quantitative and qualitative data were gathered through 241 questionnaires and the participation of 23 people in focus groups.

**Results:**

Factors found to significantly influence filter preference were duration and frequency of injecting drug use, the type of drugs injected and subculture. Furthermore, IDU's rationale for the preference of one type of filter over others was explored. It was found that filter preference depends on perceived health benefits (reduced harms, prevention of vein damage, protection of injection sites), drug retention (low retention: better high, protective mechanism against the reuse of filters; high retention: filter reuse as a protective mechanism against withdrawal), technical and practical issues (filter clogging, ease of use, time needed to prepare an injection) and believes (the conviction that a clear solution contains less active compound).

**Conclusion:**

It was concluded that the factors influencing filter preference are in favour of change; a shift towards the use of more efficient filters can be made through increased availability, information and demonstrations.

## Background

Drug preparations are commonly filtered by illicit drug users before injection in order to eliminate impurities of the drug containing solution. Several complications arising from injecting drug use depend on the characteristics of the filter used [1]. Amongst these is the introduction of insoluble particles into the blood stream. Various complications, ranging from minor to severe, are associated with the intromission of these foreign bodies. At the site of injection, sterile abscesses, cellulites and ulcers can occur, which increase the risk of infection at these sites [2,3]. After injection, insoluble particles such as talc and cellulose will stay intact and move along with the blood stream, blocking the first vessels too small to pass. Repeated administration can thus lead to severe pulmonary and cardiac complications such as talcosis [4-8]. People suffering from talcosis will experience moderate to severe dyspnoea, can develop cyanosis and even die [5,9] This condition can take one to several years to develop, but once present, the symptoms are irreversible and continue to develop despite of discontinuation of drug use [9]. Poor filtration has been suggested to be one of the risk factors for the development of talcosis [10].

All filters used by injecting drug users (IDUs) will eliminate some of these particles, but not with the same efficacy. The size of the majority of insoluble particles involved in the development of talcosis due to injecting drug use is within the range of 9 μm to 23 μm (medium of 14 μm) [11]. Cigarette filters, commonly used by injecting drug users, eliminate less than half of all particles above 10 μm [12]. Injecting drug use syringe filters (IDUSF) have been specifically conceived for drug use and are capable of eliminating the large majority of insoluble particles [13]. Three IDUSF are currently available: the Compet AG syringe filter (Compet AG, Switzerland), the "filter syringe" (Frontier Medical Group, UK) and the Sterifilt^® ^(Apothicom, France). These IDUSF are not designed to sterilize a non sterile solution, but to promote single use of filters and to eliminate particles of over 10 micron, in accordance with the European Pharmacopeia concerning injectable preparations. Besides these IDUSF, several commercial filters called wheel filters are also capable of eliminating the majority of these particles [14].

Another important characteristic of a filter is its capacity to retain drugs, as the retention of a considerable proportion of the active compound can incite drug users to hold on to their filter and to reuse it later or even to share or sell it [15]. The conservation and reuse of filters puts IDUs at risk of bacterial and fungal infections, and filter sharing is a known risk factor for hepatitis C transmission [16,17]; sharing of paraphernalia other than syringes might even be a key element in the ongoing hepatitis C epidemic. IDUSF are specifically designed to retain as little active compound as possible, without the necessity to add extra rinses to the filtration process; this is susceptible to increase their acceptability and to reduce sharing.

IDUSF can thus substantially reduce some very common injection related Public Health issues, like the incidence of small vessel blockage and subsequent medical complications [12,18].

As far as we are aware, France is the only country where an IDUSF is widely available, and free of charge, at almost all needle exchange programs (NEPs). These programs also give out sterile cotton filters (which are inside a sterile single use cooker given out as a kit, the Stericup^®^). IDUs in this country thus have the choice between two sterile filters. Besides these, some IDU use cigarette filters; other makeshift filters are rarely used in France. We describe here a study which explored filtration method preference by IDUs who have access to these three options and the influence of the drug being injected on filter preference. The characteristics of the Sterifilt^® ^are the following: it eliminates 99% of insoluble particles, and is additionally effective at shifting the particle size distribution towards the smaller range, with approximately 95% of all particles present after filtration measuring less than 5 μm [12]. This filter retains virtually no drug (0.02 ml), as opposed to cigarette and makeshift filters which retain about 0.13 ml [13].

## Methods

Two complementary methods were used to gather both quantitative and qualitative information: a questionnaire and focus groups. A total of 241 questionnaires were filled in by IDUs visiting 10 different needle exchange programs in 9 cities in France during 2009. The questionnaire explored housing, financial resources, frequency of injection, drugs injected during the last month and details on the last injection: drug injected, location, type of paraphernalia used and the reasons for using an IDUSF or not. The responses were collected by qualified drug workers after a meeting with the study lead. Focus groups or expert groups were held with the objective of obtaining detailed qualitative information on filter preference, drug preparation techniques and opinions on Sterifilt^®^. A total of 23 people in 3 different cities participated in these groups. All participants injected illicit or pharmaceutical drugs and each reported having tried the Sterifilt^® ^syringe filter at least once. Four of the participants had recently quit injection.

Quantitative analysis was conducted using EpiInfo 6. The small numbers of people using cigarette filters obliged us to group these with the cotton filters into one category: "other filters" as opposed to IDUSF use.

## Results and discussion

### Participants and drug use

The participants' main characteristics are briefly described in table 1. The group of participants under 30 contains more women (40% versus 15%), they inject more often on a daily basis (74% versus 63%) and use more heroin (75% versus 45%) and cocaine (66% versus 55%) than their older counterparts. They also live more frequently with friends and other drug users in unstable housing and less often have stable resources.

**Table 1:** Main characteristics of participants

General characteristics	Participants
Mean and median age (min-max)	31 years (16 - 53)

Female	27%

Live alone	45%

Live in a community of IDUs	14%

Receiving an income or unemployment benefit	22%

Receiving minimum social income (454,63€ per month)	42%

The four drugs or pharmaceuticals most commonly injected are: buprenorphine (Subutex^® ^and generics), heroin, cocaine and morphine sulphate (Skénan^®^) (table 2). The mean number of substances injected during the last month by individual participants is 2.5.

**Table 2:** The drugs injected by participants

Drugs injected	The last 30 days	The last injection
Buprenorphine (Subutex^® ^and generics)	63%	42%

Heroin	58%	20%

Cocaine	60%	17%

Morphine Sulphate (Skénan^®^)	39%	15%

Other	22%	6%

Cocaine users are slightly younger (median 30 versus 34) and have more precarious living conditions, they often live in unstable housing and receive low or unstable financial resources. Morphine and buprenorphine users more often inject on a daily basis. The latter ones have a longer "career" as IDUs (16 years, versus 13 for morphine users and 9 for heroin or cocaine users). Morphine users more frequently experienced a bad hit during the last 6 months (46% versus 31% for heroin or cocaine users and 19% for buprenorphine users).

### Filtration

The majority of the participants (72%) use the Sterifilt^® ^on a regular basis ("always" or "frequently") with at least one of the substances they injected during the last month. 25% use this filter regularly with all drugs injected and 43% have used this filter for their last injection. When other filters are used, these are cotton filters in 56% and cigarette filters in 33% of the cases (The remaining 11%: no filtration, commercial cotton...).

### Factors influencing filtration technique preference

Three factors were significantly associated with filter preference: the individual, the drug injected, and the city.

The syringe filter was used more often by people who inject frequently (at least 2 to 7 days a week; p < 0.001). People who started injecting when this filter was already available were more likely to use it (p = 0.02).

The choice of the type of filter used was highly correlated across drug types for given individuals, suggesting that people have the tendency to generalise their filtration technique to all the drugs they inject. However, an influence of the injected drug remains present: respondents were more likely to filter their buprenorphine with an IDUSF and their cocaine with another filter than the other way around.

As for the drugs used during the last injection, we can distinguish 3 categories amongst the 4 drugs most commonly injected in France:

The majority of the buprenorphine injectors (64%) used an IDUSF. The starch present in these tablets gives rise to several complications such as the puffy hand syndrome. Furthermore, complications at the injection site are more frequent among buprenorphine injectors [19]. Their reduction seems to be the main motive for Sterifilt^® ^use among these injectors. This is partly due to the increased extent of information flow at NEPs on the injection of buprenorphine tablets and syringe filter use.

A second category includes heroin and cocaine, for which 39% versus 33% used an IDUSF. The main argument for using syringe filters is the preservation of health; their use is facilitated by the relative lack of technical difficulties (e.g. the membrane hardly ever gets clogged by these drugs). The main arguments mentioned by this population for the continued use of cotton filters are the conservation and re-use of "old cottons" and the assumption that filtration of these drugs is less important than filtration of pharmaceuticals.

Finally, morphine sulphate capsules are almost always filtered using either cotton or cigarette filters (only 11% used an IDUSF), despite of the higher frequency of "bad hits" found to be related to their injection. This preference is due to the preparation method generally used to dissolve the morphine sulphate, which involves heating the solution before filtration, making it viscous so that even the cotton filter can be too dense to filter it.

For those who use other filters, the majority use the sterile cotton to filter buprenorphine, cocaine and heroin, while morphine sulphate injectors generally prefer a cigarette filter.

**Figure 1:**
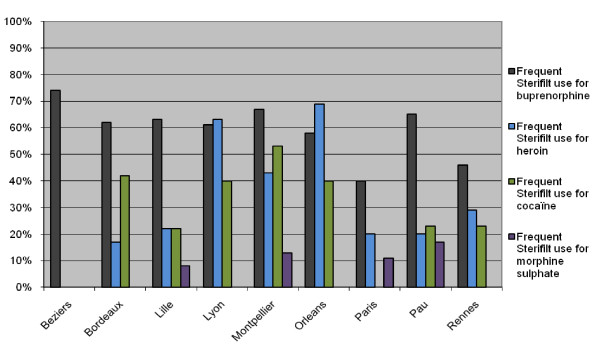
**Frequent Stérifilt^® ^use during the last month per city in relation to the drugs injected**. The percentage of IDUs who frequently ("always" or "frequent") use the Sterifilt for each of the four most commonly used drugs in 9 different French cities.

Substantial differences have been found between cities for filter preference, suggesting that local community practices may have a significant effect on filter preference (Figure 1). This is consistent with several studies which have described the importance of peer influence on drug use [15,20,21].

For buprenorphine, the cultural influence on filter preference did not show significant differences between cities (p = 0.1). These differences were quite large though (74% of the buprenorphine users in Beziers use a syringe filter, compared to only 40% in Paris), suggesting that the sample size might have been too low to obtain significance. Local filter preference differences are significant for the injection of heroin (3 levels, p < 0.0001), cocaine (3 levels, p < 0.001), and morphine sulphate (2 levels, p = 0.05).

Besides these factors, the questionnaires and focus groups revealed IDUs rationales for the preference of one filtration method over another.

### Reasons for not using a syringe filter

Most of the reasons brought up for the use of "other filters" concerned technical difficulties linked to the use of the Sterifilt^®^. Scott [13] had also observed that some IDUs find the Sterifilt^® ^difficult to use. Here, we explore which types of barriers are encountered.

Several participants mentioned that the membrane can become clogged, which is attributable to its small pore size, combined with a high density and insoluble particle content of the solution. Membrane obstruction is thus more frequent with pill injection and particularly with the injection of morphine sulphate. This is due to the method used to prepare Skénan^® ^for injection: capsules are opened and the contained microbeads are crushed, water is added and the solution heated. Due to the gelatine contained in these microbeads, the process of heating produces a dense solution which clogs the filtering membrane. A participant of the Focus group in Toulouse illustrates this very well:

*"I inject morphine sulphate every day (...). In the beginning, I heated to solution. I thus used a cotton filter, or more often a cigarette filter, because it was difficult. I used a very small piece of cigarette filter, the smallest possible, otherwise it wouldn't get through."*(male, age 26)

Preparation and filtration using a syringe filter can be slow. This can be an obstacle at two distinct moments:

When fitting the filter to the syringe, which takes some training and concentration.

*"For me it's the speed, not the speed of filtration, which I believe is fast, but to fit the filter to the syringe. (...) I go through a lot of trouble, especially when I have used a lot *(of drugs) *or when I shiver (...). You have to be very concentrated to fit the Sterifilt to the syringe"*. (male, age 41)

When pulling up the plunger. Due to the small pore size, it takes some time for the solution to get through.

*"I don't use the Sterifilt often. Filtration is slow and takes too much time. When I don't have the time, I don't filter at all" *(female, age 33)

The Sterifilt^® ^might damage the needle. If a person is in a hurry, under the influence of drugs or in withdrawal while fitting the Sterifilt^® ^to the syringe, he or she might lack the concentration needed to perform this operation. The needle might then touch the plastic of the filter and thus get damaged.

*"When I use a Sterifilt, I often damage a needle. As I don't use drugs regularly, to limit my consumption, I only take two syringes when I plan to use. If I then damage the needle, I don't have spare needles. I thus prefer using a cotton filter." *(male, age 36)

Related to these technical difficulties is the fact that IDUs don't always have the time, though some spoke about patience, to use this filter; especially if they are stressed or if they prepare their injection in a maladapted environment (public toilets, street).

*"Filtration also depends on the context. Even though you always filter as you should, this is impossible if you have to inject in public toilets."*(male, age 35)

It is interesting to note that the information from the field, which suggests that the Sterifilt^® ^is more readily adopted by IDUs who have stable living conditions than by those who live and use in the street, cannot be confirmed by the quantitative data. Though several participants, as here above, stated that some contexts are indeed less adapted than others, no correlation has been found between precarious living conditions and Sterifilt^® ^use.

As the use of a syringe filter changes the drug preparation ritual, for some, there never seems to be an appropriate moment to try out a new technique or tool. The perseverance of existing injecting practices can thus be a second major barrier to the use of syringe filters.

Several presumptions about current practices may act as a barrier to syringe filter use, such as the idea that someone's current filtration technique is efficient in eliminating particles, or that some drugs don't need filtration because no insoluble particles are visible in the solution. However, clear solutions are not always free of them, as some potentially harmful insoluble particles are invisible to the naked eye [22]. Cocaine and heroin filtration was believed to be of less importance because of the absence of tablet fillers such as starch and talc, and due to the lower prevalence of complications at the injection site (in France, complications at the injection site are more prevalent among buprenorphine users [19]).

*"I only use the Sterifilt when my coke is very filthy" *(male, age 39)

Others presume that the Sterifilt^® ^is only meant for frequent injectors, a presumption reflected by the fact that frequent injectors use this filter more often.

*"I don't use the Sterifilt often. I know it is better, but I don't use drugs often, not daily. It is important for guys who inject every day" *(male, age 36)

Furthermore, some IDUs who inject an opaque solution for years can experience considerable difficulties admitting that their drug is completely water-soluble and that a transparent solution thus contains the same amount of active compound.

*"The cotton is more efficient, for the solution is white" *(female, age 25)

Additionally, similar to the findings of Scott [13], IDUSF's low drug retention can be considered as a disadvantage. Several respondents reported retaining cotton filters as a means to keep a small amount of drug "for later" and did not wish to change this behaviour. This conservation and reuse of cottons was associated with the injection of heroin, morphine sulphate and cocaine. These drugs are difficult to obtain and expensive; their consumption can be compulsive and/or shortage can readily induce withdrawal. Reuse of cottons was rarely mentioned by buprenorphine users.

*"I always used the Sterifilt solely for Subutex *(buprenorphine) *(...). For heroin and cocaine, I had that old "craze" to use a cotton and keep it (...). I did it all: squeeze the last drop out of the filter using my hands, everything." *(female, age 32)

A final barrier to the use of IDUSF was that they are not readily available everywhere. Though virtually all NEPs in France give them out, they are not accessible at syringe vendor machines or at pharmacies where 60 to 80% of all syringes are exchanged [23,24]. This excludes IDUs who do not visit NEPs from being familiar with this filter, and influences its acceptance and habituation by other IDUs, as they do not have access to it at each time they exchange syringes.

*"New injectors don't go to needle exchange programs (...). Habits from the beginning persist; Sterifilt should be given to new ones" *(male, age 36)

### Reasons for using a syringe filter

The two main arguments reported by IDUs for using the Sterifilt^® ^are the quality of filtration and more generally the role of this filter in the prevention of health problems.

Participants reported that solutions filtered by this IDUSF contained less "chunks", were cleaner and clearer. Buprenorphine users were particularly likely to describe these as advantages of syringe filters. Additionally, buprenorphine users mention specifically the removal of starch as an advantage of syringe filters.

*"Less deposit, less starch, cleaner" *(male, age 30)

Secondly, similar to the findings of Scott [13], participants either state that they believe the Sterifilt^® ^reduces drug related harm or that they have actually experienced a reduction in complications such as abscesses, bad hits, and phlebitis.

*"I've had a phlebitis, that's the reason why I adopted the Sterifilt. Since, I don't have "pins and needles in my legs" anymore, less abscesses and no more phlebitis" *(male, age 36)

Respondents also refer to the capacity of this filter to preserve the injection site. They state that when the solution is unintentionally injected into the soft tissue surrounding the vein, complications are less harmful and of a shorter duration.

*"I use the Sterifilt to get rid of particles. When I miss my hit *(the expression "a missed hit" refers to the deposit of the solution outside the vein), *I don't have any abscesses any more" *(male, age 48)

Furthermore, veins are described to recover more quickly when a syringe filter is used.

*"Veins recover when you use the Sterifilt. You can't use it *(the vein) *for a couple of days, but after that, you can reuse it" *(male, age 39)

This may be due to the reduction in the number of large insoluble particles in the solution and/or to the protection of the needle by the filter when it is appropriately fitted to the syringe. Indeed, this syringe filter covers the needle completely and thus prevents it from touching the inside of the cooker.

Most Sterifilt^® ^users consider its low drug retention as positive: they gain a better high because there is virtually no loss of active compound. This characteristic can also serve a protective role, removing the temptation to keep filters for later. Indeed, for some, even though they are aware of the risks and do not want to reuse filters, it is difficult to throw away a filter containing 6 to 13% of the active compound. This tendency was also described by Scott [13].

*"You gain in active product and you won't do the cotton" *(male, age 26)

Finally, several people familiar with its use described the Sterifilt^® ^as easy and quick to use.

It is interesting to note that IDUs never mentioned the prevention of hepatitis C transmission through the reduction of reuse and sharing as a reason for IDUSF use. It seems that, for individual users, local complications and vein damage are of more immediate concern, as they occur almost instantly after injection. As for pulmonary complications, most people are unaware of the link between injecting drug use, filtration methods and pulmonary problems, and may impute respiratory difficulties to infections or tobacco use.

## Conclusion

IDUSF and other commercial syringe filters have the capacity to considerably reduce harms associated with injecting drug use, such as complications at the injection site, and pulmonary problems due to the injection of insoluble particles, but also hepatitis C transmission through the reduction of reuse and sharing of filters due to their reduced drug retention. Several studies [13,14] have evaluated the efficacy of IDUSF or wheel filters. Scott's research [13] also included some questions on filter preference. However, to our knowledge, this is the first study exploring the factors and reasons for filter preference among injecting drug users in detail. A better understanding of factors and IDUs' motives influencing filter choice may help drug workers to promote the use of less harmful filters. The unique French situation of high availability of IDUSF and sterile cotton filters creates the opportunity to study these factors.

The results show three main factors influencing the filter of choice:

Individual drug users have the tendency to generalise their preparation method to all drugs used. Additionally, people who started injecting when the IDUSF filter already was available, use it more often, as do people who inject frequently.

The drugs injected. Three categories can be distinguished amongst the four drugs most commonly injected:

The majority of the buprenorphine injectors (64%) used an IDUSF during their last injection, mainly to reduce the frequent complications at the injection site related to the injection of this drug.

39% of the heroin users and 33% of the cocaine users used the syringe filter to preserve their health. The cotton filter on the other hand, is often preferred for it allows "keeping some for later" in order to prevent withdrawal; furthermore, fine filtration of these drugs is often considered less important.

Only 11% of the people injecting morphine sulphate used this filter, due to frequent technical difficulties, like membrane obstruction, which is, in its turn, related to the preparation method used for this drug.

Subculture and peer influence on preparation- and harm reduction techniques were confirmed by a variation in local filter preference.

These three factors suggest that positive change is possible: influence of the individual and subculture, as well as the relatively slow but stable progression of syringe filter use, suggest that these behaviours can potentially be changed.

Harm minimization information will probably be more efficient if it addresses the advantages perceived by IDUs. Once understood, technical difficulties may become relatively less important. This study shows that the reduction of complications at the injection site is perceived as the main advantage of syringe filters. The transparency of the solution, the reductions of abscesses and cellulites, as well as the preservation of veins are often referred to by drug users. The gain in active compound due to low drug retention also seems to be an important issue which, in addition, can be used as a protective mechanism against the re-use and sharing of filters.

However, barriers remain for some users. It is necessary to acquire the technique to use this type of filter, filtration is relatively slow and the filter membrane may be clogged by cutting agents and tablet fillers. It is thus important for needle exchange programs to provide frequent demonstrations, accompanied by the diffusion of appropriate information and prevention messages on the solubility of drugs and the harms associated to the injection of insoluble particles. The provision of information has been effective in France, where information flow was concentrated on buprenorphine use; the majority of its users have adopted the IDUSF. Increased availability would probably also enhance IDUSF use.

Previous research has shown that drug users are preoccupied by their health and willing to change their behaviour. To change preparation and filtration techniques, information should be concentrated on the perceived advantages of new techniques; these will be more convincing and able to promote change.

## List of abbreviations

IDU: Injection Drug User; IDUSF: Injecting Drug Use Syringe Filter; NEP: Needle Exchange Program.

## Competing interests

Elliot Imbert is the inventor of the Sterifilt^® ^and the main stockholder of Apothicom Distribution. Lenneke Keijzer works at Apothicom, organisation which developed and sells the Sterifilt^® ^as well as the cotton filter. This research was set up and conducted by Apothicom in order to obtain a better understanding on filtration method preferences in a country where several filters are readily available; information which might be useful to adapt Apothicom's services or tools to IDUs practices.

## Authors' contributions

LK and EI conceived and designed the study; LK implemented the study design, including data collection. LK performed the statistical analysis, wrote the manuscript and coordinated the revisions. Both authors revised the manuscript and read and approved the final draft.
